# Cancer patients’ needs for volunteer services during Covid-19: a mixed-method exploratory study

**DOI:** 10.1186/s40359-023-01453-3

**Published:** 2023-12-01

**Authors:** Sara Alfieri, Laura Gangeri, Simonetta Sborea, Claudia Borreani

**Affiliations:** 1 Clinical Psychology, Fondazione IRCCS Istituto Nazionale dei Tumori, Via Venezian 1, Milan, 20133 Italy; 2 Lega Italiana per la Lotta contro i Tumori (LILT), Milan, Italy

**Keywords:** Covid-19 pandemic, Mixed methods, Patients’ needs, Oncology, Volunteer services

## Abstract

**Introduction:**

To date, there are no known studies that have investigated the new need for volunteer services among cancer patients during the Covid-19 pandemic. However, it is essential for volunteer associations to heighten such knowledge to best guide their offer in this challenging period.

**Aim:**

The present study aims to provide a mapping of the cancer patients’ needs for volunteer services followed at Istituto Nazionale dei Tumori in Milan (Italy) during the Covid-19 pandemic. Since there are no specific questionnaires for this purpose, we created an ad hoc tool for which we report the preliminary result.

**Method:**

We used a mixed-method multiphase approach. Phase I: in April-May 2020 40 ad hoc paper questionnaires were distributed at the entrance of the aforementioned hospital, with the aim of investigating patients’ needs through two open-ended questions then analyzed through thematic analysis. Phase II: the contents that emerged from Phase I were transformed into items and submitted to the judgment of a small group of “peers” (patients) and “experts” (professionals) in November-December 2020 to evaluate their comprehensiveness, representativeness and intrusiveness. Phase III: in January-February 2021 paper questionnaires, containing the items reviewed in Phase II, were distributed within the hospital to a representative sample of cancer patients. We applied descriptive statistics, Exploratory Factor Analysis (EFA) and Cronbach’s Alpha.

**Results:**

32 patients completed Phase I, 3 “peers” and 9 “experts” participated in Phase II, 214 patients completed the questionnaire in Phase III. EFA highlights five kinds of needs during the Covid-19 pandemic, in order of priority: (1) need to be supported at the hospital; (2) need for emotional support; (3) need for daily errands; (4) need for practical support to family members; (5) need to share free time. Preliminary results on the tool are encouraging, although further studies are needed. These results will allow local volunteer associations to adapt their services during the pandemic.

**Supplementary Information:**

The online version contains supplementary material available at 10.1186/s40359-023-01453-3.

## Introduction

The Covid-19 pandemic has had a devastating impact, not only in healthcare systems [1–3] but also in the economic, financial, political, and educational spheres [4–7]. While a great deal of attention has been paid to the impacts of the pandemic on the economic and healthcare systems, less attention has been given to unpaid activities, such as volunteer work [8, 9].

Volunteering is a form of social action that refers to people’s prosocial behaviors of carrying out activities freely and free of charge for the benefit of others [10], within an organization [11]. In 2019, based on the latest pre-Covid ISTAT data [12], in Italy 336.275 active non-profit institutions employed a total of 5 million 529 thousand volunteers and 788 thousand employees. Their contribution is fundamental because volunteers are usually engaged in activities and fields that are not well supported by the market or the government [13–15], such as the areas of education, environment and health [16, 17]. According to Connors [18], volunteers also fill many gaps in hospital systems, mostly in times of staffing shortages. Pre-pandemic studies show that volunteers can positively influence the quality of care for both patients and caregivers by reducing stress levels and offering practical and emotional support as well as providing links to the community [19–22]. Volunteers offer their attention and time in supporting patients and their caregivers when employees or nurses may not be available. Although families provide most of the necessary care to patients, volunteers take on important roles, for example offering practical and emotional support to reduce stress and providing a link to the community, etc. [5, 6]. Some studies have found that the volunteers’ engagement to perform complementary contributions in hospitals is a cost-effective method that increases positive patient satisfaction [23, 24]. Vanderstichelen et al. [35] reported that some patients define volunteers as the “other face of care”, emphasizing the ease with which patients confide in people who offer them psychological, social, and existential care. Therefore, according to the authors, volunteers represent a “liminal space” between cancer patients and the healthcare system.

Hence, the presence of volunteers is even more crucial to respond to cancer patients’ needs [25–27]. In existing literature, there is a lack of knowledge regarding cancer patients’ needs for volunteer services specifically before the Covid-19 pandemic. Instead, more extensive literature is available regarding cancer patients’ needs in general terms, which ranges from clinical and financial issues to emotional support to employment and legal issues (such as minimum fee exemption and recognition of disability) [26, 28–36]. Some of these studies have used a qualitative approach (e.g. [33, 35]) to explore all possible needs, without, however, providing a ranking of those of highest priority. Some exceptions can be seen in [29] and [30]. In these studies, the need for illness and treatment-related information unanimously emerged as the first need [29, 30, 33]. Psychological and social supports were found to be important but not priorities and are generally placed in second place [29, 30, 33, 35]; less frequently reported needs are economical and legal support [35] and, even less frequently, are practical needs [29, 30, 35]. Instead, they were unanimous in affirming that many needs such as psychological support remain unsatisfied [26, 30, 33]. In research focusing on the needs of cancer patients conducted in Italy [33] before the pandemic, the need for cooperation between associations and social and health services emerged. This research showed how these organizations are often disjointed and compete with each other, their services being fragmented as well. Furthermore, cancer patients’ need to be informed about social assistance, monetary support, legal and work protection emerged, from social workers’ and health and care assistants’ perspectives.

The Covid-19 pandemic determined some changes in the volunteer sector, such as: 1) the reduction of the amount and quality of services provided [8, 37] due to the impossibility of carrying out activities in settings in which these organizations usually operate (e.g., hospitals) or due to the impossibility of pursuing their purposes (e.g., carry out public awareness initiatives); 11) a decline in the numbers of volunteers [38]; 111) changes regarding the “mode” in which activities are delivered: many activities had to change from “in-person” to “online”; 1 V) some activities, which were typically well “organized and structured”, gave rise to “more spontaneous” forms of volunteer services influenced by the nature of extreme urgency [37].

In Italy, as in many other European countries, during the Covid-19 pandemic, the Government asked people to reduce any kind of social contact, promoting the slogan “stay at home”, because only by strictly complying with the isolation measures was it possible to respond adequately to the pandemic challenges. Nevertheless, Italy was one of the first countries to be most affected by the Covid-19 pandemic, in terms of both number of deaths [39, 40] and economic impact [4]. The Government choice - albeit necessary - led to the cessation of most of the volunteer activities, even those in hospitals, care homes and hospices. However, as previously reported, volunteer services are crucial for both patient care and a better functioning of the healthcare system [41].

Many studies of the volunteer field were conducted during the pandemic (e.g. [8, 37, 38, 42, 43]). However, no known studies have analyzed the specific needs of the people to whom volunteer services are directed. In particular, no studies have investigated the needs for volunteer services by patients who must be cared for by reference hospitals, such as cancer patients. Oncological disease impacts people’s lives as it significantly changes physical, psychological and social balances [44–48]. During the Covid-19 pandemic, patients with chronic illnesses had to cope with their pathology [49], as well as the feeling of vulnerability and an increase in stress, anxiety and depression levels [50]. In such a vulnerable time, many cancer patients were able to enjoy the help and support of hospital volunteers.

To respond to recent research calls about the impact of Covid-19 on the volunteer sector [51], this study aims to investigate the needs of cancer patients from the Fondazione IRCCS Istituto Nazionale dei Tumori in Milan (INT), needs that can be satisfied by volunteer organizations. Understanding the needs of cancer patients during the Covid-19 pandemic is fundamental for volunteer associations, because in this type of historical moment, they have to necessarily change their actions in line with needs and restrictions which have never existed before.

Unfortunately, there are also no pre-Covid studies conducted in our Comprehensive Cancer Centre on cancer patients’ need for volunteer services. However, a quantitative study conducted in our hospital in 2003 on hospitalized cancer patients’ general needs [29] revealed that among the five requests expressed most frequently by cancer patients, four regarded information needs: concerning diagnosis, future conditions, a better dialogue with clinicians and about economic-insurance information. Support-assistance needs were less reported. The needs that were less frequently expressed were “practical” ones, as help to eat, dress, and visit the bathroom.

## The present study

This study is part of a larger project called “Volontariato 3.0” [Volunteering 3.0] [52], an action-research [53] project that aims to: (i) analyze challenges and new needs of volunteer organizations, patients and healthcare facilities of the hinterland of Milan; (ii) promote actions to increase volunteer services to respond to the emerging needs of the above mentioned actors during the pandemic; (iii) give support and training to the volunteer organizations involved to address the pandemic by putting all available resources in place. Within the wider project “Volontariato 3.0”, this mixed method study aims primarily to investigate the needs of cancer patients from the Fondazione IRCCS Istituto Nazionale dei Tumori (INT) in Milan (Italy) that could have been satisfied by volunteer organizations during the Covid-19 pandemic. Since there are no specific questionnaires for this purpose, we created an ad hoc tool for which we report the preliminary results.

To achieve the research aims, the mixed methods approach was the most appropriate choice. This approach has the potential to respond to the aim of the research as it incorporates everyday, pragmatic languages (qualitative) as well as technical and representative (quantitative) data [54]. As affirmed by Sale et al. [55] (p.44): “based on their paradigmatic assumptions, the two methods do not study the same phenomena. Evidence of this is reflected by the notion that quantitative methods cannot access some of the phenomena that health researchers are interested in, such as lived experiences as a patient, social interactions, and the patients’ perspective of doctor-patient interactions.” For this reason, this study consists of three phases: (I) qualitative, which carries out a recognition of all possible needs; (II) qualitative and quantitative, which consists of the development of a tool aimed at investigating cancer patients’ needs for volunteer services; (III) quantitative, which prioritizes and synthesizes patients’ needs.

Appendix 1 shows the chronogram of the research phases throughout the development of the Covid-19 pandemic.

### The pandemic scenario inside the hospital

All three phases of the study were conducted at the Fondazione IRCCS Istituto Nazionale dei Tumori (INT), a Comprehensive Cancer Centre in Northern Italy (Milan). The INT, foundation, and government-designated centre for treatment and research, is a leading cancer centre pursuing mainly clinical and translational research, exploring and developing the fields of biomedicine and public health, in order to deliver high quality healthcare services. It has been designated a Comprehensive Cancer Centre by the Organization of European Cancer Institute (OECI) in recognition of its excellence both in patient care and the development of new treatments. Its research aims to improve prevention, early diagnosis and treatment of cancer disease, as well as the quality of life of cancer patients.

Within the hospital, there are numerous associations that provide constant assistance to the patient (e.g., giving them directions to the hospital entrance, accompanying them to medical visits, proposing recreational and artistic courses within the hospital, etc.), to caregivers (e.g., giving them directions, entertaining them while waiting for visits, etc.) and to health personnel (e.g., carrying out errands, performing administrative functions, etc.). From the end of February 2020 up to the beginning of January 2021, to reduce and regulate gatherings, the INT Management prohibited access to all volunteers and all patients’ relatives, in this latter case except for specific cases (e.g., underage or non-self-sufficient patients). This created a lot of disorientation in patients, especially the elderly, who had to give up both the support of their families and that of volunteers.

## Phase I

### Aims

Phase I aimed to provide an initial mapping of the needs for volunteer services among cancer patients from the INT during the Covid-19 pandemic. In line with the mixed-method approach, the rationale for Phase I was to try to obtain as complete a list as possible of these patients’ needs.

### Method

#### Participants and procedures

In the period between April 2020 and May 2020, 40 ad hoc paper questionnaires were distributed by researchers at the hospital entrance of INT. All hospital out-patients were considered eligible. The exclusion criteria were: (i) being a minor; (ii) not speaking the Italian language fluently.

#### Measures

The questionnaire included 2 “open” questions: (1) “*If you could have a volunteer at your disposal, how do you think he/she could help you?*”; (2) “*Are there any places or moments in your life when you might need the help of a volunteer the most?*“. To speed up the compilation and not weigh the questionnaire down, the socio-demographic variables were not asked. The questionnaire was administered in an anonymous way.

#### Analyses

The answers to the two “open” questions were analysed together through “paper-pencil” thematic analysis [56], with the aim of identifying some themes (or categories). Themes are quotations capable of capturing important semantic concepts useful for answering research questions [56]. The analyses were conducted using an inductive (bottom-up) approach, which means that the themes derive from the content of the quotations themselves and are identified by researchers during analysis [56, 57]. Based on the contents that emerged, these themes were turned into a list of needs. Redundant responses were eliminated. The analyses were conducted jointly by two researchers [SA and LG], who are experienced in qualitative research. All disagreements were addressed from time to time and agreement was always reached.

### Results

#### Participants

32 completed questionnaires (80% compliance rate) were returned. Some patients did not return the questionnaire upon exit, declaring that they were in a hurry, while others declared that they did not have time to fill it out before the visit or the analyses. The number of respondents is in line with the number expected from a qualitative research [58, 59] and, furthermore, the theoretical saturation [60] was reached at the 25th questionnaire.

#### Thematic analysis

From content analysis, 39 different needs emerged, ranging from the need to have a volunteer to spend time with (e.g., to do pleasant activities together, to be able to chat, etc.), to ask for small errands (e.g., to go shopping or at the pharmacy), to provide information within the hospital, to help understand how to find financial aid, etc. The complete list of needs is presented in Table 1.

**Table 1:** List of items/needs that emerged in Phase I

List of items/needs that emerged
*A volunteer who…*
1. … keeps me company during hospitalisation
2. … reassures my relatives about my physical and psychological conditions when I can’t see them
3. … goes food shopping in my place
4. … accompanies me to check up or treatments when I am inside the hospital
5. … comes to my house and takes me to check ups or treatments
6. … gives me information at the entrance of the hospital
7. … gives me information about my clinical path
8. … carries out little errands (to go to the post office or pharmacy, to go and buy the newspaper, to take the dog for a walk, etc.)
9. … keeps me company at home
10. … listens to me when I need it
11. … helps me to buy post-operative materials
12. … helps me economically when I buy post-operative materials
13. … helps my relatives with accommodation
14. … helps my relatives to orient themselves outside the hospital
15. … helps me in web activities at home
16. … comes with me to cultural and recreational events
17. … helps me with drainages at home
18. … teaches me how to use a laptop, tablet or smartphone at home
19. … makes me lunch when I’m not well
20. … helps me in caring for the children when I’m not well
21. … helps my children with homework when I’m not well
22. … carries out little errands for my relatives when I can’t
23. … keeps me company during hospitalisation
24. … is available to speak by video call
25. … takes a walk with me
26. … greets me when I arrive at hospital
27. … helps me to understand the doctor’s directions better
28. … shares his/her passions (for ex. Needlework, board games, etc.) with me
29. … reads aloud to me
30. … helps me with housekeeping
31. … stays at home with me after operations
32. … gives me information about the path I have to take
33. … is the middle-man between health staff and me
34. … gives me emotional support
35. … keeps me company in the evenings
36. … comes with me to do food shopping
37. … helps me to understand my rights as patient
38. … helps me to organise my free time
39. … will be there for me when they unplug the machines that keep me alive

## Phase II

As to date, there are no known tools which could have detected the needs for volunteer services among cancer patients during the Covid-19 pandemic, Phase II was structured to build a tool for detecting those needs. The rationale for Phase II was to produce a tool that is understandable, relevant, and not offensive or overly intrusive.

### Method

#### Participants and procedures

To achieve the aim of the research, the procedure suggested by Chiorri [61] for the construction of new measuring instruments was implemented. This procedure requires the involvement of a limited number of “peers” (in our case, cancer patients) and “experts” (in our case, professionals working with cancer patients and leaders of volunteer associations) in evaluating content validity. This procedure is considered necessary in order to obtain an external and authoritative perspective that can help researchers in technical aspects such as: the identification of the items to eliminate, to reformulate, the length of the instrument, etc. [61].

In the period between November 27, 2020 and December 29, 2020, 15 questionnaires (5 for “peers” and 10 for “experts”) were distributed to participants. The patients were recruited among those present at the Department of Clinical Psychology in two established recruitment days; the professionals were chosen from among the collaborators of Department of Clinical Psychology of the hospital (other than researchers) and included the leader of Lega Italiana per la Lotta contro i Tumori (LILT) Association.

#### Measures

The needs that emerged in Phase I were transformed into items and included in a questionnaire. For each item, participants were asked to rate three aspects on a Likert scale ranging from 1 (= *not at all*) to 5 (= *very much*): comprehensiveness, representativeness, and intrusiveness. Furthermore, participants were asked, if they deemed it necessary, to make changes to the items when delivering back the questionnaire and to report any aspect they wished.

#### Analyses

Following the procedure suggested by Lynn [62], the content validity was calculated. For each item, a content validity indicator was calculated individually for the three aspects investigated (comprehensiveness, representativeness, and intrusiveness) and overall. To be considered satisfactory, values had to be between 0.80 and 1.00 [62]. All comments made by “peers” and “experts” were discussed jointly by two researchers [SA and LG] as to whether to accept the proposed changes or not.

### Results

#### Participants

3 patients (“peers”; 60% of compliance rate) and 9 professionals (“experts”; 90% of compliance rate) answered the questionnaire within the established time frame. The characteristics of the participants are shown in Table 2.

**Table 2:** Description of the participants (“peers” and “experts”) in Phase II

	Sex	Age range	Profession	Level of education
**“Peer”**				
1	Female	30–39	Housewife	Master degree
2	Female	30–39	Teacher	Bachelor degree
3	Female	50–59	Employee	High school diploma
**“Expert”**				
4	Female	40–49	Social worker	Master degree
5	Female	20–29	Volunteering employee	Master degree
6	Female	50–59	Psychologist	Bachelor degree
7	Female	40–49	Volunteering coordinator	Bachelor degree
8	Male	40–49	Psychotherapist Psychooncologist	Master degree
9	Female	60–69	Psychotherapist Psychooncologist	Master degree
10	Female	60–69	Nursing and Psychooncologist	Master degree
11	Female	60–69	Responsible for the volunteering area	High school diploma
12	Female	30–39	Psychotherapist Psychooncologist	Master degree

#### Content analysis

The results of content validity are shown in Table 3. Most items (n = 23) meet the criteria suggested by Lynn (1986). Items that were not satisfactory (n = 16) were modified following the recommendations of peers and experts. Seven items were eliminated because they were considered not relevant (e.g., *“Someone who helps me with drainages at home”*) or intrusive (e.g., *“Someone who will be there for me when they unplug machines that keep me alive”*). Therefore, the final list consists of 32 item/needs.

**Table 3:** Results of the validity of content carried out by “peers” and “experts”

Item n.	Comprehensibility	Representativeness	Intrusiveness*	Total
1	0.88	0.85	0.90	0.88
2	0.83	0.81	0.67	**0.77**
3	0.92	0.73	0.91	0.85
4	0.94	0.79	0.81	0.85
5	0.94	0.94	0.96	0.95
6	0.92	0.9	0.92	0.91
7	0.85	0.79	0.65	**0.76**
8	0.94	0.79	0.81	0.85
9	0.94	0.79	0.92	0.88
10	0.85	0.79	0.88	0.84
11	0.79	0.73	0.65	**0.72**
12	0.77	0.63	0.65	**0.68**
13	0.81	0.81	0.83	0.82
14	0.77	0.71	0.90	**0.79**
15	0.71	0.60	0.88	**0.73**
16	0.88	0.52	0.94	**0.78**
17	0.85	0.60	0.77	**0.74**
18	0.90	0.73	0.98	0.87
19	0.88	0.79	0.77	0.81
20	0.90	0.75	0.79	0.81
21	0.88	0.67	0.75	**0.77**
22	0.77	0.6	0.69	**0.69**
23	0.95	0.86	0.95	0.92
24	0.92	0.85	0.9	0.89
25	0.90	0.75	0.92	0.86
26	0.90	0.88	0.90	0.89
27	0.90	0.75	0.79	0.81
28	0.92	0.63	0.92	0.82
29	0.96	0.75	0.94	0.88
30	0.96	0.75	0.85	0.85
31	0.85	0.77	0.75	**0.79**
32	0.81	0.50	0.69	**0.67**
33	0.88	0.38	0.56	**0.61**
34	0.67	0.58	0.67	**0.64**
35	0.83	0.83	0.83	0.83
36	0.83	0.83	0.83	0.83
37	0.75	0.75	0.75	0.75
38	0.67	0.67	0.67	**0.67**
39	0.58	0.58	0.58	**0.58**

Notes: * Intrusiveness scores have been reversed: low scores indicate low intrusiveness and high scores indicate high intrusiveness. The unsatisfactory values have been reported in bold

## Phase III

### Aim

Phase III aims at a two-fold objective: (1) to prioritize and synthesize the needs that emerged from Phase I and were formulated through Phase II. To do so, it is necessary to have a tool that aims to do so and is formulated with clear questions and is representative of the investigated topic and non-intrusive phenomenon; (2) therefore, we propose to present preliminary results of a tool which was specifically created for this purpose.

### Method

#### Participants and procedures

In the period between January 21, 2021 and February 8, 2021, 200 paper questionnaires were distributed at the entrance of the INT. On December 30, 2020, following the worsening of the pandemic situation in Italy, a link containing an online version of the questionnaire was sent to all members of the Palinuro association’s through the Google Moduli platform.

All hospital out-patients were considered eligible. The exclusion criteria were: (i) being a minor; (ii) not speaking the Italian language fluently.

#### Measures

Based on the results that emerged in Phases I and II, a questionnaire consisting of 32 items/needs was prepared. Also, an item with the wording “other” and the possibility of adding a text for explanation were added. Respondents were asked to indicate on a 5-step Likert scale (*1 = not at all* to *5 = very much*) how important each item/need was to them.

#### Analyses

In order to prepare a list of priorities in needs, the means (M) and standard deviations (SD) of each item were calculated. For each item, the 95% confidence interval (CI) was also provided. To group the items by type and, therefore, to have indications about the synthesis, we performed Exploratory Factor Analysis (EFA). Gerbing and Hamilton [63] suggest that EFA has to be used prior to any analysis technique to confirm hypotheses on data structure. We used Principal Axis Factoring with Oblimin Rotation, which is the extraction method most widely used in literature [64]. We have also shown communality which indicates the percentage of explained variance of each item.

To verify homoscedasticity, the Bartlett test - which must be statistically significant - was calculated. The Kaiser-Meyer-Olkin was also used to measure sampling adequacy. To be considered acceptable, values must be higher than 0.70.

Cronbach’s Alpha (α) is used to measure the internal consistency of the dimension. Values above 0.70 are considered acceptable, 0.80 or greater is preferred [65].

All the analyses were carried out using SPSS software V. 26.0.

### Results

#### Participants

214 patients answered the questionnaire. Among them, 84.1% completed the questionnaire in paper format. 53.3% were male, with a mean age of 58.17 years (range 19–90; SD = 14.82); 22.7% had an elementary or middle school diploma, 44.4% a high school diploma, 31.4% a degree (three or five years), 1.4% answered “other”. 50.3% said they went to the hospital for a check-up, 21.4% for therapy, 8.6% for a consultation, 19.8% answered “other” (e.g., booking an appointment or delivering reports). Finally, 88% declared that they were patients exclusively of INT, the others said that they were receiving care in other hospitals as well.

#### Priority of needs

Among the item/needs perceived as priorities are: to receive information at the hospital entrance (M = 4.06), the possibility for the volunteer to reassure family members, who cannot enter the hospital, about the state of health of patients (M = 3.93) and, also, to receive information about their rights (M = 3.69). The means, SD and 95% CI of each item are shown in Table 4, in order of priority. Appendix 2 shows the items in the original language (Italian) and the English translation.

**Table 4:** Means, Standard Deviation and Confidence Intervals for each item

How much of a priority is it for me to have a volunteer who…			95% CI	
	M	*SD*	Lower	Higher
….gives me information at the entrance of the hospital	4.06	*0.84*	3.71	4.35
….encourages my relatives about my physical and psychological condition when I can’t see them	3.93	*1.05*	3.29	4.14
….helps me to understand my rights as a patient	3.69	*1.16*	3.42	4.18
….helps me to understand my doctor’s directions better	3.58	*1.20*	2.91	3.84
…. accompanies me to check up or treatments when I am inside the hospital	3.50	*1.18*	2.96	3.84
… helps me to buy materials suggested by doctors (e.g. bras, colostomy bags, etc.)	3.48	*1.09*	3.28	4.09
…listens to me when I need it	3.47	*1.20*	3.06	3.97
… greets me when I arrive at hospital	3.45	*1.22*	3.09	3.93
….gives me emotional support	3.42	*1.28*	2.68	3.61
… keeps me company during hospitalisation	3.31	*1.10*	2.97	3.71
… comes to my house and takes me to check ups or treatments	3.26	*1.35*	2.68	3.78
… helps my relatives to orient themselves outside the hospital	3.14	*1.38*	2.48	3.47
…helps my relatives with accommodation	3.09	*1.44*	2.37	3.40
… goes food shopping in my place when I need it	3.02	*1.31*	2.76	3.69
…suggests me who can help me economically	2.94	*1.37*	2.41	3.36
…carries out little errands (to go to the post office or pharmacy, to go and buy the newspaper, to take the dog for a walk, etc.)	2.87	*1.25*	2.53	3.42
….makes me lunch when I’m not well	2.73	*1.33*	2.23	3.08
….is available to speak by video call	2.72	*1.27*	2.52	3.37
… keeps me company at home after discharge	2.71	*1.35*	2.32	3.22
…. keeps me company at home	2.70	*1.30*	2.24	3.07
…. carries out little errands for my relatives when I can’t	2.70	*1.31*	2.29	3.19
…helps me with online activities when I’m at home (e.g. connect by laptop,tablet or smartphone, communicate with other people, use technological platforms, etc.)	2.65	*1.36*	2.20	3.17
… helps me in caring for the children when I’m not well	2.65	*1.53*	1.78	2.79
… takes a walk with me	2.55	*1.30*	2.22	3.15
… comes with me to do food shopping	2.49	*1.29*	1.96	2.90
… teaches me how to use a laptop,tablet or smartphone	2.44	*1.40*	1.93	2.92
…. helps me with housekeeping	2.39	*1.30*	2.08	2.95
… keeps me company in the evenings	2.39	*1.28*	1.85	2.78
… helps me to organise my free time	2.38	*1.24*	1.91	2.78
… shares his/her passions (for ex. Needlework, board games, etc.) with me	2.37	*1.18*	2.12	2.97
… takes me to cultural and recreational events	2.34	*1.22*	2.35	3.30
… reads aloud to me	2.22	*1.22*	1.85	2.67
Other (specify________________________________)	2.09	*1.38*	1.01	2.56

#### Synthesis and articulation of needs

EFAs were performed to identify the number of factors that emerged. The solution consists of 32 items that saturate 5 different dimensions, for a total explained variance of 67.03%, which is satisfactory. We defined these factors as follows: 1) need to share free time (which explains most of the variance: 52.41%); 2) need to be supported in the hospital (6.28% of variance explained); 3) need for practical support to family members (3.17% of variance explained); 4) need for daily errands (2.96% of variance explained); 5) need for emotional support (2.20% of variance explained). As Table 5 shows, items 9, 17, 18, 19, 28, 30 saturate several factors at the same time, which indicates that these items belong to multiple factors.

**Table 5:** Factor loading, Cronbach alpha and Percentage of variance explained emerged from the EFA

		Factors					
		1	2	3		4	5
		Need to share free time	Need to be supported at the hospital	Need for practical support to family members	Need for daily errands		Need for emotional support
25… shares his/her passions (e.g., sewing, board games, etc.) with me		**0.825**	0.045	0.104	− 0.086		− 0.056
22… takes walks with me		**0.799**	0.106	− 0.058	0.115		− 0.004
26… reads aloud to me		**0.792**	− 0.012	0.192	− 0.123		− 0.001
21… is available to speak by video calls		**0.774**	0.073	− 0.233	0.030		0.180
20… keeps me company at home after discharge		**0.717**	0.006	− 0.112	0.160		0.189
32… helps me to organise my free time		**0.680**	0.166	0.099	− 0.056		0.086
27… helps me with me with the household chores		**0.663**	− 0.064	0.150	0.182		0.010
15… takes me to cultural and recreational events		**0.657**	0.067	0.200	0.203		− 0.212
16… teaches me how to use laptop, tablet or smartphone		**0.596**	0.047	0.191	0.154		− 0.012
8… keeps me company at home		**0.572**	0.026	− 0.152	0.424		0.157
14… helps me with online activities when I’m at home (e.g., laptop, tablet or smartphone, communicate with other people, use technological platform, etc.)		**0.490**	0.064	0.222	0.235		− 0.021
17… makes me lunch when I’m not well		**0.484**	0.098	**0.325**	0.131		0.052
30… comes with me to do grocery shopping		**0.478**	0.073	0.106	**0.318**		0.093
29. keeps me company in the evenings		**0.461**	0.169	0.124	0.194		0.179
11… suggests me who can help me economically		**0.314**	− 0.073	0.241	0.159		0.203
6… gives me information at the entrance of the hospital		− 0.179	**0.945**	− 0.043	0.091		− 0.087
23… greets me when I arrive at the hospital		0.007	**0.814**	0.022	− 0.015		− 0.048
24… helps me understand my doctor’s directions better		0.224	**0.671**	0.002	− 0.069		0.111
31… helps me to understand my rights as a patient		0.136	**0.583**	0.114	0.029		0.070
10… helps me to buy materials suggested by doctors (e.g., bras, colostomy bags, etc.)		0.112	**0.550**	0.111	0.022		0.039
4… accompanies me to check ups or treatments when I am at the hospital		0.055	**0.410**	− 0.049	0.312		0.249
13… helps my relatives to orient themselves outside the hospital		0.003	0.153	**0.704**	0.102		0.075
12… helps my relatives to find accommodation		0.110	0.034	**0.678**	0.052		0.140
18… helps me to care for the children when I’m not well		**0.389**	0.050	**0.409**	0.027		0.207
19… carries out little errands for my relatives when I can’t (e.g., go to the post office, grocery shopping, etc.)		0.270	0.060	**0.356**	**0.323**		0.000
5… comes to my house and takes me to check ups or treatments		0.033	0.125	0.012	**0.682**		0.173
3…. shop for groceries when I need it		0.085	0.037	0.182	**0.658**		0.077
7… carries out little errands (e.g., to go to the post office or the pharmacy, to go and buy the newspaper, to take the dog for a walk, etc.)		0.221	0.145	0.205	**0.584**		− 0.063
2… encourages my relatives about my state of physical and psychological health when I can’t meet them		− 0.082	− 0.090	0.121	0.138		**0.696**
28… gives me emotional support		0.231	**0.414**	0.079	− 0.241		**0.475**
1… keeps me company during hospitalisation		0.069	0.260	0.073	− 0.038		**0.431**
9… listens to me when I need it		**0.326**	0.157	− 0.181	0.131		**0.395**
*Cronbach Alpha*	*0.969*		*0.881*	*0.857*	*0.892*		*0.751*
Percentage of variance explained	52.41		6.28	3.17	2.96		2.20

The Kaiser-Meyer-Olkin Measure was found to be 0.94 and indicates that the sample is optimal to perform the EFA. Bartlett’s test was statistically significant, χ^2^ (496) = 4004.17, *p* < .001, which demonstrated the presence of homoscedasticity, and so the variances of the factors can be compared. All communalities of items had satisfactory values (between 0.41 and 0.78), thus indicating that all items were sufficiently “strong” to be considered for EFA.

All values of Cronbach’s Alpha were largely satisfactory. This confirms that items grouped in a dimension measure are coherent in meaning [65].

**Fig. 1:**
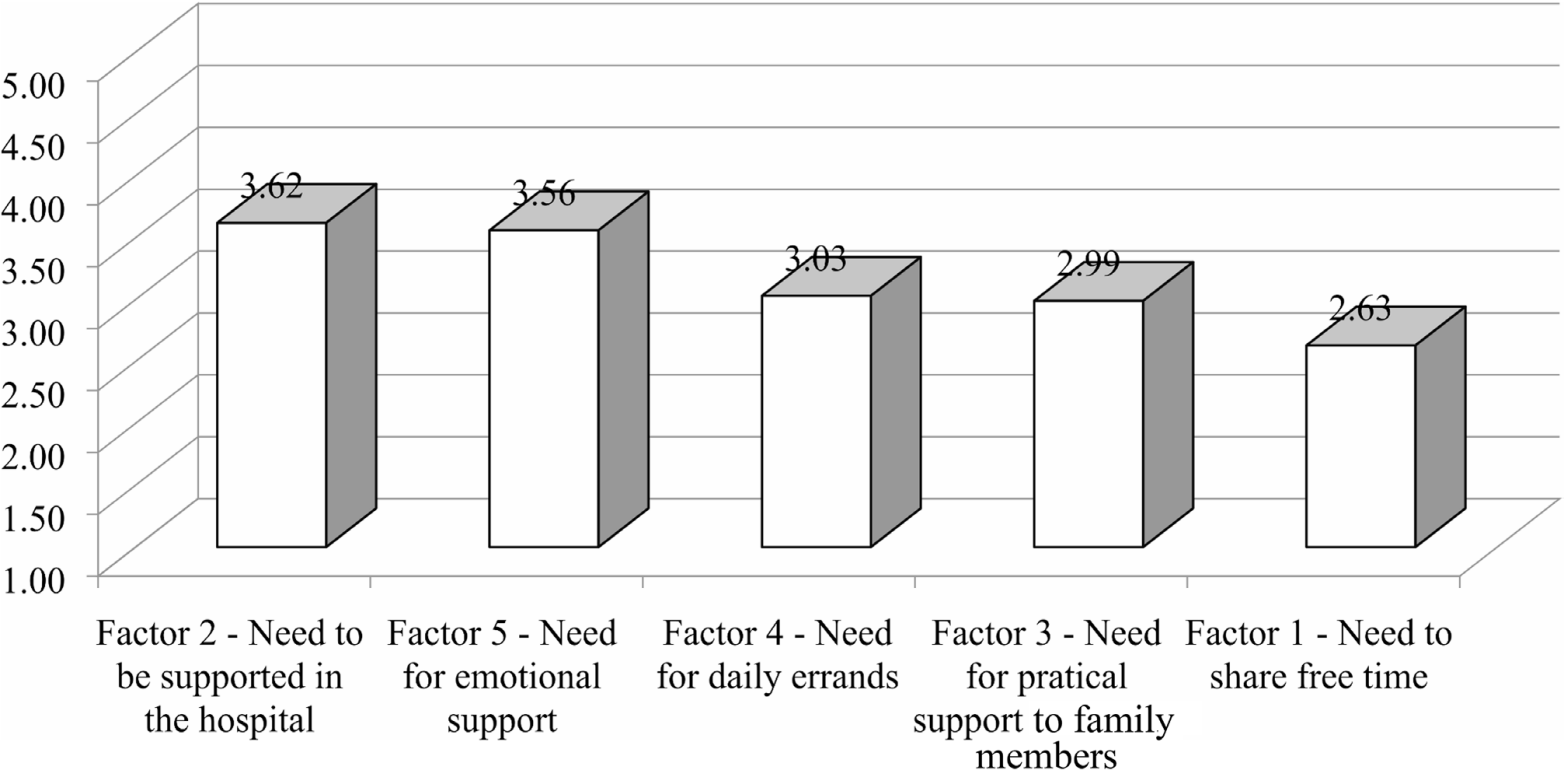
Means of the five factors that emerged from EFA

Figure 1 shows the means of the five factors that emerged, in order of priority. All factors are correlated with each other, in particular: Factor 1 (Need to share free time) and 3 (Need for pratical support to family members); Factor 1 and 4 (Need for daily errands) (Table 6). This means that the five factors measure similar, but not equal, properties.

**Table 6:** Correlations between the components that emerged from the PCA

Component	1	2	3	4	5
1. Need to share free time	-	0.690	0.781	0.758	0.619
2. Need to be supported at the hospital		-	0.574	0.582	0.644
3. Need for practical support to family members			-	0.669	0.497
4. Need for daily errands				-	0.504
5. Need for emotional support					-

Notes: All correlations are significant at *p* < .01

## General discussion

Our study aimed at opening a reflection on the needs of volunteer services among cancer patients during the Covid-19 pandemic. Looking at single items, the most important items/needs for cancer patients were represented by the opportunity for them to refer to a volunteer who gives them information at the hospital entrance, reassures family members about their state of health, as well as helping them understand their rights as patients and, helping them understand the indications given by doctors. The least important items/needs are those linked to the need to share free time. Knowing these priorities promptly was crucial for the “Volontariato 3.0” project, as it provided answers to the world of associations both about new activities to be implemented and to those to be modified to meet INT cancer patients’ needs.

The EFA results highlighted five kinds of needs, which will be presented in order of priority (please also see Fig. 1 for a summary). The first and the highest priority is the *need to be supported at the hospital* (which includes items such as “…gives me information at the entrance of the hospital”, “…accompanies me to check up or treatments when I am inside the hospital”, etc.), which encompasses the needs of volunteer services that involve “being patient”. This group is very important, because it closely concerns the needs that patients feel inside the hospital where they are treated. In our opinion, this type of need was particularly felt during the Covid-19 pandemic as all the volunteers who helped patients to find their way around the hospital were unable to carry out this service, leaving these needs uncovered. In line with what emerged from other research [18], the presence of volunteers within the hospital is crucial for the hospital functioning itself, as volunteers support functions that doctors, nurses and administrative staff, overloaded with a lot of commitments, cannot perform. In our Comprehensive Cancer Centre more than 200 volunteers have been constantly present for over 25 years. Over this time, they have become valuable reference points for patients. Therefore, their absence during the pandemic was probably perceived by patients as an additional source of isolation and confusion, which was added to that produced by Covid-19. Since this is a set of needs that does not emerge in other research, we cannot know whether these needs were present before the pandemic or not. It is possible to assume that they were already present, but were never detected, due to a lack of research on this topic.

The second need in terms of priority refers to the *need for emotional support* (which includes items such as “…gives me emotional support” and “encourages my relatives about my physical and psychological condition when I can’t see them”, etc.), which concerns the need for attention to the personal and family emotional sphere. This result is in line with some pre-pandemic studies that identified this particular need as one of the most pressing [26, 30, 33, 35]. This result is also in line with studies that, in the last two years, highlighted a feeling of vulnerability, an increase in stress, anxiety and depression in the general population due to the Covid-19 pandemic [50, 66]. Cancer patients, who face the disease and the pandemic simultaneously, may be more vulnerable than the general population. In addition, many hospitals have postponed some non-urgent visits, increasing the sense of insecurity and abandonment in these patients.

The third need refers to the *need for daily errands*, concerning patients’ need to have support for small errands (e.g., shopping) or for transport needs (e.g., a volunteer who accompanies them to the hospital). The need for transport is not a surprise, due to the feeling of distrust towards public transport (often crowded and dirty) during the Covid-19 pandemic. However, this is a result that only a few projects have highlighted. Few pre-pandemic studies revealed these types of needs [29, 30, 35]. It must be pointed out, however, that Tamburini et al’s paper [29] referred to inpatients, whereas ours are all out-patients.

The fourth need refers to the *need for practical support to family members* (which includes items such as “…helps my parents to orient themselves outside the hospital”, “…Carries out little errands for my relatives when I can’t (e.g., go to post office, go shopping, etc.”) – which concerns the respondent’s need for a volunteer who can take care of his/her family members when he/she is in the hospital (for hospitalization, for treatment, etc.). In this regard, it should be specified that the INT is a national reference hospital for oncological pathologies, and therefore a lot of patients go there even though they reside in other regions, which sometimes, are very distant. In addition, due to restrictions caused by the Covid-19 pandemic, caregivers/carers couldn’t enter hospitals. This implied that caregivers/carers sometimes had to find accommodation even for medium-long periods, and they had to orient themselves in an unknown and very large city like Milan.

The fifth and the lowest priority need refers to the *need to share free time* (i.e., “…takes a walk with me”, “…helps me to organize my free time”, “…keeps me company in the evenings”, etc.) that includes all aspects of people’s need for a volunteer who can keep them company, share hobbies, teach small recreational techniques, etc. at home. These needs, too, were not reflected in pre-pandemic literature, probably as they were caused by it. Before the pandemic, people were not forced to “stay at home” and to isolate socially (except for a few rare cases of social marginalization), so companionship and sharing hobbies were usually performed by family members, friends, neighbours, etc.

Factors 1 and 3 and 1 and 4 are quite correlated. This result could indicate that those who “Need to share free time” also have more “Need for practical support to family members” and “Need for daily errands” (and vice versa, since there is a correlation). This may suggest more needs need to be met.

Unfortunately, there are no studies carried out before the Covid-19 pandemic on the needs for volunteer services among cancer patients, and therefore, it is not possible to understand what needs increased during it. However, it is conceivable that the pandemic accentuated and made some needs, that were already present before it, more evident (need for emotional support), and brought out others that, in pre-pandemic studies, did not emerge (need to be supported inside the hospital, need to share free time, need for daily errands, need for practical support to family members).

In conclusion, then, our results are partially in line with those found in literature on the needs of cancer patients before the pandemic. In particular, the need for information of a different nature was already present before the pandemic itself, although not specific about volunteer services, as it referred to health and treatment issues [29, 33]. The information needs that emerged from our research are predominantly located in the context of orientation within the hospital, as patients feel “lost” and “without reference points”.

Psychological and social support needs were also present before the pandemic, despite some studies [29, 30, 33, 35] highlighting how they were subordinate to the needs for information. In our study, however, they ranked second place. It is worth noting that this type of need, along with those grouped with free time sharing, are the only ones that can be transformed from “in-presence” to “online”, while the rest necessarily require a new reorganization of volunteer activities from associations.

Regarding the need for daily errands, existing literature [29, 35] revealed how these were present *within* hospitals, inpatients, or hospices. However, it is interesting to notice that in our study the need for information *inside* the hospital adds up to practical needs *outside* the hospital, once patients return home.

Finally, previous studies [29] had already highlighted needs for financial and legal support, which also emerged from our study.

Regarding the psychometric properties of the ad hoc tool we provided, the results are encouraging, although they should be considered preliminary and further studies are needed. “Peers” and “experts” judged the tool to have a good content validity and made important suggestions for it to be further improved. The EFA gave satisfactory results in terms of factorial articulation (confirmed by the internal consistency found through Cronbach’s Alpha), while the saturation of single items on each factor can be improved either by eliminating some items or by changing their word formulation. However, to complete the validation process, further steps are needed: primarily, a Confirmatory Factor Analysis (CFA). Since there are no known instruments that aim for similar purposes, it is not currently possible to assess convergent validity.

The needs of volunteer services among cancer patients are important for research, policy and practice. The results of our research are interesting both from an operational point of view, as they would have allowed local voluntary associations to adapt their services during the pandemic; and also from a research point of view, as they bring to light an issue that has been poorly investigated before, during and after the pandemic.

### Limitations

This study has some limitations which have to be taken into account. The first and most important limitation is the lack of surveys of the needs for volunteer services among cancer patients before the Covid-19 pandemic within our hospital. If it had been possible to make a comparison between before and during the pandemic, we would have been able to understand quantitatively which needs have increased and which have not changed. Secondly, in Phase I the socio-demographic data of the patients who filled out the questionnaire were not collected. The choice not to propose socio-demographic questions was made to speed up the compilation time and not increase the time spent in the hospital. The negative perception of hospitals during the pandemic and the fear of being infected right in them [67–69] is worth a mention. While, on one hand, this made it possible to speed up the compilation by out- patients who entered the hospital, on the other hand, it did not allow for information about the characteristics of those who actually filled it in. Thirdly, Phase III participants were small in number. However, during the pandemic period, a lot of patients were advised not to go to the hospital except for urgent reasons, and many visits were changed to web-based meetings. For privacy reasons, it was not possible to use the email addresses present in the institutional database, and only those from the lists of the INT association could be used. Finally, the tool derived from the present study, despite showing some promising results, needs to be used carefully. In fact, before we can talk about “validation”, further steps are needed: for example, the administration of the questionnaire to a larger sample, a CFA, checking the discriminant validity, etc.

Future studies will be able to monitor changes in the needs of cancer patients over time to observe whether and how they modify in the post-pandemic period and foresee possible reorganization in the light of this transformation.

### Electronic supplementary material

Below is the link to the electronic supplementary material.

**Supplementary Material 1: Appendix 1** - Chronogram of the research phases in the development of the Covid-19 pandemic. **Appendix 2** - Italian and English versions of the items used

## Data Availability

All the material is available from the corresponding author for reasonable requests.
