# Supplementary material for: Cancer patients’ needs for volunteer services during Covid-19: a mixed-method exploratory study
Source: BMC Psychol. 2023 Dec 1;11:421. doi: 10.1186/s40359-023-01453-3 (PMC10691064; doi:10.1186/s40359-023-01453-3)
Supplement: Supplementary file 1 — Supplementary Material 1: Appendix 1 - Chronogram of the research phases in the development of the Covid-19 pandemic. Appendix 2 - Italian and English versions of the items used [file 40359_2023_1453_MOESM1_ESM.docx]

Appendix 1 - Chronogram of the research phases in the development of the Covid-19 pandemic.

| **Study phases** | **Months** | **Covid-19 Phases in Italy** |
| --- | --- | --- |
|  | February 2020 | Warning of the first cases of Covid-19 in Italy.  INT prohibits access to all volunteers |
| Detection of the problem by Volunteer Associations and study design | March 2020 | WHO declares the state of pandemic. The Italian Government shuts down Lombardy first and then the rest of Italy. Lombardy becomes a “red zone”. Intensive Care Units in a state of collapse. |
| **Phase I**  Start administration of the questionnaire | April 2020 | Lockdown continues in Italy. |
| **Phase I**  End of the administration of the questionnaire | May 2020 | The Italian Government announces the beginning of “phase 2”, which predicts the restart of some work activities and the possibility to move within one’s region. |
| **Phase I**  Data Analysis | June 2020 | Phase 3 begins, which increases reopening and provides easing of restrictive measures. |
|  | July 2020 | INT allows a small number of volunteers to carry out triage activities at the hospital entrance (control of body temperature and correct use of masks) |
|  | August 2020 | The curve of contagiousness rises once more, and restrictions begin again. |
|  | September 2020 | The virus rebounds in many European countries, but seems stable in Italy. Schools are being reopened. |
|  | October 2020 | Beginning of the second wave in Europe. |
| **Phase II**  Start administration of the questionnaire | November 2020 | Italy is divided into zones again. New lockdown and “red zone” in Lombardy. The critical threshold of intensive care beds exceeded by far. |
| **Phase II**  End of the administration of the questionnaire and data analysis | December 2020 | To prevent travel during the holiday season, all of Italy becomes a “red zone”. At the end of December, the first cycle of vaccinations begins. |
| **Phase III**  Start administration of the questionnaire | January 2021 | The vaccination campaign continues, but first virus variants begin to circulate.  Gradual restart of the activity of INT volunteers. |
| **Phase III**  End of the administration of the questionnaire | February 2021 | Italy changes government; Mario Draghi takes office as Prime Minister. The third wave begins and the Italian Government issues new restrictive measures, including closing schools. |
| **Phase III**  Data Analysis | March 2021 |  |
| Feedback of results to the voluntary associations and start of the restructuring of the proposed activities | April 2021 | The curve of infections starts to slow down in Italy, pressure on hospitals decreases. At the end of the month, Italy returns to the “yellow zone”. |
|  | May 2021 | The contagion curve continues to fall, but the Delta variant is discovered. |

*Notes:* INT: Istituto Nazionale dei Tumori, Milan, Italy.

Source: Il sole 24 ore. *Cose che noi umani* [*Things that we humans*]. Available at [La storia del coronavirus dall’inizio (ilsole24ore.com)](https://lab24.ilsole24ore.com/storia-coronavirus/?refresh_ce=1). Accessed the 4th of April 2023.

Appendix 2 - Italian and English versions of the items used.

| ***Quanto è per me prioritario avere un/a volontario/a che…***  **How much of a priority is it for me to have a volunteer who…** |
| --- |
|  |
| *…mi dia informazioni all’ingresso dell’ospedale*  …gives me information at the entrance of the hospital |
| *…rassicuri i familiari circa il mio stato di salute fisica e psicologica, quando non li posso incontrare*  …encourages my relatives about my state of physical and psychological health when I can’t see them |
| *…mi aiuti a capire i miei diritti in quanto malato/a*  …helps me to understand my rights as a patient |
| *…mi aiuti a comprendere meglio le indicazioni che mi ha dato il medico*  …helps me understand my doctor’s directions better |
| *…all’interno dell’ospedale mi accompagni a fare le visite o le terapie*  …accompanies me to check ups or treatments when I am inside the hospital |
| *…mi dia informazioni su dove acquistare i materiali suggeriti dai medici (ad es. reggiseni, sacchetti per stomie, ecc.)*  …helps me to buy materials suggested by doctors (*e.g.*, bras,colostomy bags, etc.) |
| *…mi ascolti quando ho bisogno*  …listens to me when I need it |
| *…mi accolga all’ingresso dell’ospedale*  …greets me when I arrive at the hospital |
| *…mi fornisca supporto emotivo*  …gives me emotional support |
| *…mi tenga compagnia durante la degenza in ospedale*  …keeps me company during hospitalization |
| *…mi venga a prendere a casa e mi porti a fare le visite o le terapie*  …comes to my house and takes me to check ups or treatments |
| *…aiuti i miei parenti ad orientarsi al di fuori dell'ospedale*  …helps my relatives to orient themselves outside the hospital |
| *…aiuti i miei parenti a trovare un alloggio*  …helps my relatives to find accommodation |
| *…vada a farmi la spesa quando ne ho bisogno*  …shop for groceries when I need it |
| *…mi indichi chi mi può aiutare economicamente*  …suggests me who can help me economically |
| *…vada a fare delle piccole commissioni (ad es. andare in posta o in farmacia. comprare il giornale, portare fuori il cane, ecc.)*  …carries out little errands (*e.g.*, to go to the post office or the pharmacy, to go and buy the newspaper, to take the dog for a walk, etc.) |
| *…mi prepari il pranzo quando non mi sento bene*  …makes me lunch when I’m not well |
| *…sia disponibile a parlare tramite videochiamate*  …is available to speak by videocalls |
| *…mi faccia compagnia a casa dopo le dimissioni*  …keeps me company at home after discharge |
| *…mi faccia compagnia a casa*  …keeps me company at home |
| *…aiuti i miei familiari quando io non posso farlo per piccole commissioni (ad es. andare in posta, a fare la spesa, ecc.)*  …carries out little errands for my relatives when I can’t |
| *…mi supporti nelle attività su internet quando sono casa (ad es. a collegarmi con il portatile,tablet e smartphone a comunicare con altre persone, utilizzare piattaforme tecnologiche, ecc.)*  …helps me with online activities when I’m at home (*e.g.*, using laptop,tablet or smartphone, to communicate with other people, use technological platforms, etc.) |
| *…mi aiuti nella cura dei figli quando non mi sento bene*  …helps me to care for children when I’m not well |
| *…venga con me a fare delle passeggiate*  …takes walks with me |
| *…venga con me a fare la spesa*  …comes with me to do grocery shopping |
| *…mi insegni ad utilizzare PC o smartphone*  … teaches me how to use PC or smartphone |
| *…mi aiuti con le faccende domestiche*  …helps me with the household chores |
| *… mi tenga compagnia la sera*  …keeps me company in the evenings |
| *…mi aiuti ad organizzare il mio tempo libero*  …helps me to organize my free time |
| *…condivida con me le sue passioni (ad es. cucito, giochi di società, ecc.)*  …shares his/her passions (*e.g.*, sewing, board games, etc.)with me |
| *…mi accompagni ad eventi culturali o ludici*  …takes me to cultural and recreational events |
| *…legga per me a voce alta*  …reads aloud to me |
| Other (specify________________________________)  *Altro (specificare________________________________)* |
